# Energy Landscape-Guided Virtual Screening of Side-Chain Engineering in Polymer Dynamics Design

**DOI:** 10.3390/polym17172298

**Published:** 2025-08-25

**Authors:** Han Liu, Sen Meng, Liantang Li

**Affiliations:** 1 United Laboratory of Advanced Electrical Materials and Equipment Support Technology, Electric Power Research Institute, China Southern Power Grid (CSG), Guangzhou 510663, China; mengsen@csg.cn; 2 SOlids inFormaTics AI-Laboratory (SOFT-AI-Lab), College of Polymer Science and Engineering, Sichuan University, Chengdu 610065, China; lltslz@163.com

**Keywords:** graft polypropylene, chain mobility, molecular dynamics simulation

## Abstract

Side-chain engineering is versatile for tuning the chain mobility of graft polymers and governs their thermal stability. However, it remains elusive to predict the graft effect on chain mobility, especially for competitive side-chain types. Here, relying on molecular dynamics simulation and energy landscape theory, we introduce a three-stage virtual pipeline to sequentially refine the screening of graft chain mobility while minimizing computation cost, by taking the example of grafting similar side-chain types (hydroxyethyl methacrylate (HEMA), methyl methacrylate (MMA), and vinyl acetate (VAC)) onto amorphous polypropylene (PP). Ascribed to their structural similarity, these graft systems exhibit a non-evident chain mobility distinction, with the atom displacement—governing the local “roughness” in potential energy landscape (PEL)—exhibiting only weak-to-modest correlation with their initial atomic energy, volume, and stress. This necessitates the subsequent-stage screening for broader PEL navigation, which confirms a stability and roughness rank of VAC ≥ MMA > HEMA > PP, with their chain activation energy revealing that these side chains enhance the PEL roughness through a counterbalance between possibly lowering the overall energy barrier but extensively wrinkling the landscape. Overall, the three-stage screening establishes a state-of-the-art efficient strategy to evaluate thermal stability of graft polymers in stepwise higher precision from local to ergodic roughness inspection.

## 1. Introduction

Polymer grafting is a strategic approach to modify the dynamical properties of pristine polymer materials and invent graft polymers with tailored atom dynamics and chain mobility that governs polymer thermal stability [1,2,3]. By grafting side chains onto the polymer backbones, the graft chain dynamics would shift to either a slower or faster relaxation mode based on the side-chain types [4,5,6]. Considering the numerous types of side-chain modifications, this side-chain engineering requires deliberate consideration in determining the side-chain type by priorly revealing the graft effect on chain mobility [7]. However, the relationship between the graft structure and its chain dynamics remains largely unexplored and elusive, rendering it challenging to predesign a side-chain modification satisfying various thermal stability scenarios of graft polymers [8,9,10].

Owing to its in silico efficiency [11], molecular dynamics (MD) simulation provides a unique opportunity for virtual screening of graft chain mobility and offers an atomistic insight into the graft effect [12,13,14,15]. Recent MD studies have revealed that graft chain dynamics are closely correlated to their side-chain-length- and conformation-induced steric effect [14,15]. These outcomes greatly facilitate and deepen our understanding of structure–property relationship of graft polymers. Nevertheless, in the case of competitive side chains that share a similar chemical structure, their graft effect on chain dynamics remains nearly untapped, except for a few experimental evaluations [7,8]. Although similar side chains generally lead to less apparent dynamics distinctions, these graft polymers such as certain graft polypropylene polymers might somehow exhibit immense societal significance and are required for evaluation in various thermal stability scenarios [16,17,18]. However, without a deliberate MD protocol for virtual screening, this structural similarity likely results in an undistinguishable or unreliable nuance in chain dynamics.

From the viewpoint of potential energy landscape (PEL) [19,20,21,22,23,24], polymer chain dynamics are governed by the landscape roughness and augmented by a higher energy barrier and curvature, thus lowering atom mobility and stabilizing chain dynamics [25,26,27]. Herein, the PEL roughness is defined as the inverse of PEL topography flatness and is quantified by atom mobility in the local topography. Several MD-based metrics have been proposed to evaluate the roughness-governed chain mobility [28,29,30,31], including (i) system-level metrics, e.g., glass transition temperature [32], characteristic relaxation time [15], mean squared displacement [33], diffusion coefficient [34], mechanical stiffness [35], etc., and (ii) local-level metrics, e.g., atom displacement [36], atom Voronoi volume [37], atom softness [38], etc. These metrics ranges across thermodynamic, dynamic, structural, and mechanical quantities to characterize the energy landscape roughness and the resultant atom mobility in relaxation or deformation mode, estimating either local or more ergodic roughness based on the PEL inspection scope, which is tuned by various MD simulation protocols involving timescale and temperature [28,39]. However, the different MD protocols and mobility metrics would lead to a wide spectrum of computation costs and precision resolution in evaluating the PEL roughness, the graft chain mobility and, eventually, the polymer thermal stability [30]. This wide spectrum of precision resolution arises from the fact that (i) the high-dimensional wrinkled energy landscape is too complicated to depict precisely with a wide distribution of local roughness and (ii) the resultant chain dynamics are too complex to predict precisely by intuitive structural and thermodynamic metrics [40,41]. As such, there is a lack of MD protocols to rationalize virtual screening of graft chain dynamics in optimal precision resolution while minimizing the computation cost.

Here, relying on MD simulation and PEL theory [20,21], we introduce a three-stage virtual pipeline to sequentially screen side-chain types and evaluate polymer thermal stability, with stepwise higher computation costs and precision from local to ergodic roughness inspection of PEL, with the finest precision resolution to distinguish chain mobility at each temperature for polymers grafted by similar competitive side chains, by taking the example of grafting three similar side-chain types (hydroxyethyl methacrylate (HEMA), methyl methacrylate (MMA), and vinyl acetate (VAC)) onto amorphous polypropylene (PP) (see Section 2). All three-stage screenings adopt atom displacement at the local level and, statistically, mean squared displacement at the system level, to characterize graft chain mobility, considering the fact that the atom motion trajectories are the elementary basis for chain dynamics analysis [40], wherein the trajectory itself constitutes a minimal computation cost but ensures precise estimation of the local PEL roughness at the finest resolution at each temperature.

Stage 1 screening essentially estimates one local roughness using atom displacement of one equilibrated system (see Section 3.1), which is suitable for identifying competitive side-chain types from numerous candidates at a minimal computation cost. In addition, it is notable that the atom displacement shows only weak-to-modest correlation with their initial atomic energy, volume, and stress (see Section 3.2), which suggests that the complex dynamics governed by PEL roughness is unable to make predictions using these simple intuitive non-dynamical quantities, thus necessitating more precise dynamics-based roughness estimation in subsequent-stage screening. Stage 2 screening essentially quantifies the roughness distribution by statistically averaging the atom displacement in several parallel configurations sampled across the PEL (see Section 3.3 and Section 3.4), which yields a largely overlapped roughness distribution for the graft PP systems herein. Stage 3 screening further disentangles the roughness overlap by significantly broadening the scope of PEL inspection from local to ergodic roughness estimation based on chain dynamics at elevated temperatures and extended timescales (see Section 3.5 and Section 3.6). Herein, the Arrhenius-activation computation of the atom diffusion coefficient provides an optimal precision resolution of PEL roughness at each temperature (see Section 3.7) and, synergically, yields an overall roughness rank of VAC ≥ MMA > HEMA > PP, wherein their diffusion activation energy reveals that these side chains enhance the PEL roughness through a counterbalance between possibly lowering the overall energy barrier but extensively wrinkling the landscape by increasing the overall curvature of energy basins. Overall, this work paves the way to evaluate graft chain dynamics and polymer thermal stability in silico, toward precise yet efficient side-chain engineering.

## 2. Methods

### 2.1. Preparation of Various Graft Polypropylene Systems

**Figure 1:**
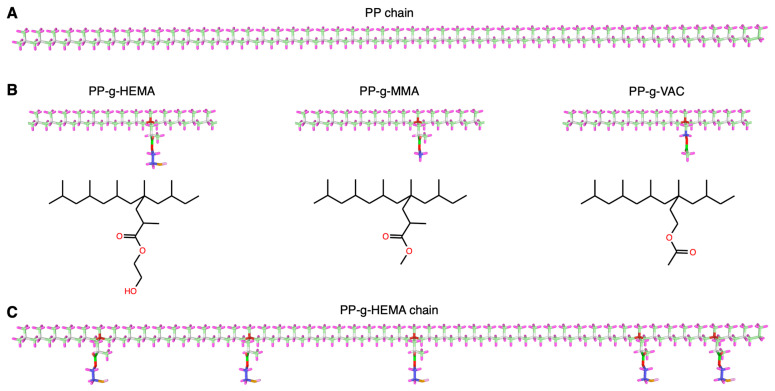
Graft polypropylene (PP) chain preparation. (**A**) A single PP chain configuration consisting of 50 monomers. (**B**) Three types of graft PP chains. The side chains are set as hydroxyethyl methacrylate (HEMA), methyl methacrylate (MMA), and vinyl acetate (VAC), respectively. By convention, the graft PP chains are termed PP-g-HEMA, PP-g-MMA, and PP-g-VAC, respectively. (**C**) A graft PP chain configuration with 5 HEMA side chains, i.e., a PP-g-HEMA chain herein.

**Figure 2:**
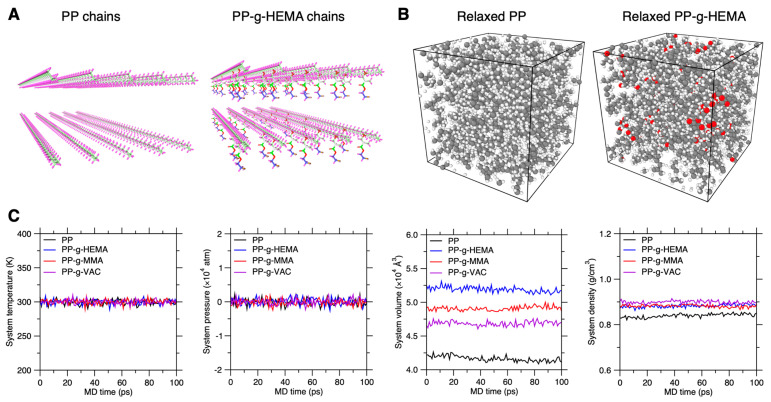
Graft PP chain relaxation by MD simulation. (**A**) Initial configuration of the graft PP systems consisting of 10 chains each, i.e., the PP system and PP-g-HEMA system herein. (**B**) Equilibrium configuration of the graft PP systems relaxed under NPT ensemble. (**C**) System temperature, pressure, volume, and density of the equilibrated systems recorded for a relaxation duration of 100 ps.

We first prepare the initial configurations of graft polypropylene (PP) systems. The PP chain consists of 50 monomers (see Figure 1A) and is prepared by the Moltemplate package [42]. The bond length and angle are set based on their geometric optimum. Then, three types of side chains are randomly grafted onto the PP backbone chain in the same sequence pattern, including hydroxyethyl methacrylate (HEMA), methyl methacrylate (MMA), and vinyl acetate (VAC). By convention, the formed graft PP chains are termed PP-g-HEMA, PP-g-MMA, and PP-g-VAC, respectively (see Figure 1B), and the graft rate of each chain is herein fixed to 10%, i.e., five side chains per backbone (see Figure 1C). All initial configurations consist of 10 identical graft PP chains by duplication, and the chains are parallelly placed into a cubic simulation box with a side length of 135 Å to fully encompass the straight chain conformation (see Figure 2A), which is then relaxed to an amorphous condensed state under an NPT ensemble (see Section 2.2). Note that the scope of this research solely investigates the amorphous region of semicrystalline PP systems, which exhibits significantly more active chain dynamics than their crystalline counterpart, thus playing a dominant role in tuning polymer thermal stability [43].

### 2.2. MD Simulation of Graft Polypropylene by COMPASS Forcefield

We then conduct MD simulations to equilibrate the graft PP configurations by the LAMMPS package [44]. The interatomic interactions in these polymer configurations are described by the well-established COMPASS forcefield [45], which has been demonstrated to offer an accurate structural and dynamical description of polymer systems in excellent agreement with experimental results [46]. The COMPASS forcefield consists of pairwise energy *U_pair_* and molecular energy *U_mol_*, as described in Equation (1) [45]:

(1) $$U=U_{pair}+U_{mol}=\left(U_{vdwl}+U_{coul}+U_{long}\right)+(U_{bond}+U_{angle}+U_{dihedral}+U_{improper}),$$

wherein the pairwise energy *U_pair_* is a summation of van der Waals energy *U_vdwl_*, short-range Coulombic energy *U_coul_*, and long-range Coulombic energy *U_long_*, and the molecular energy *U_mol_* is composed of bond, angle, dihedral, and improper energy terms denoted by *U_bond_*, *U_angle_*, *U_dihedral_*, and *U_improper_*, respectively. The detailed formulation of these component energy terms can be found in Ref. [45], and the forcefield parameters are determined using the published COMPASS parameters based on the Refs. [45,47]. The global cutoff for all pairwise interactions is set to 10 Å based on Ref. [45]. Note that the van der Waals energy computation adopts the sixth-power mix-pair interaction rule and the long-tail correction energy term [45], and the long-range Coulombic energy is computed by the Ewald summation method [48].

The initial graft PP configurations are first subjected to energy minimization, followed by the relaxation simulation under the NPT ensemble at 300 K and 1000 atm for 100 ps to reach an equilibrium state, wherein the initial pressure is to compress the system to a condensed state. The temperature of 300 K is well above the PP system’s glass transition temperature to activate chain segment dynamics [18]. The timestep for all simulations is fixed to 1 fs. Subsequently, the system gradually releases the internal stress from 1000 atm to zero pressure in 100 ps. Afterwards, we further equilibrate the system at 300 K and zero pressure for 100 ps to obtain the equilibrated configuration (see Figure 2B). Finally, this equilibrated configuration is used as the starting configuration to record atom motion trajectories under the NPT ensemble at 300 K and zero pressure for 100 ps (see Section 3.1 and Section 3.2). And the evolution of system temperature, pressure, volume, and density with time is recorded in Figure 2C, which is nearly constant to indicate the equilibrium state of the graft PP systems. Moreover, the pristine PP configuration exhibits an equilibrium density around 0.835 g/cm^3^, very close to experimental references [16,18], which posteriorly validates the simulation precision.

Note that to conduct configuration-averaged statistical analysis of chain dynamics recordings (see Section 3.3 and Section 3.4), another four parallel start configurations are prepared by sampling configurations from an elevated-temperature trajectory under the NVT ensemble at 500 K, conducting energy minimization, and re-equilibrating the configurations at 300 K for 20 ps under the NPT ensemble before the atom trajectory recording. Moreover, to compute the atom diffusion coefficient at different temperatures from 300 to 500 K (see Section 3.5 and Section 3.6), we re-equilibrate the start configuration at each temperature for 20 ps under the NVT ensemble and, afterward, record atom motion trajectories in the NVT simulation up to 1 ns. Finally, to estimate the glass transition temperature of graft PP systems (see Section 3.7), the start configuration is first subjected to an equilibration simulation at an elevated temperature of 400 K for 20 ps under the NVT ensemble, followed by recording the evolution of thermodynamic quantities of the system in a cooling simulation under the NVT ensemble from 400 K to 200 K in 100 ps. The Nose-Hoover thermostat and barostat are used to adjust the system temperature and pressure for all simulations [49].

### 2.3. Atomic Quantities Computation of Equilibrated Polypropylene

During the recording of atom motion trajectories, we simultaneously compute some relevant atomic quantities of the equilibrated systems using the LAMMPS and OVITO packages [44,50], including atom displacement, atom potential energy, atom Voronoi volume, and atom normal and shear stress [33,35,38]. In detail, the atom displacement, *d_i_*, is computed by Equation (2):

(2) $$d_{i}=\;{\left\|{\vec{r}}_{i}\left(t\right)-{\vec{r}}_{i}\left(0\right)\right\|}_{2},$$

wherein ${\left\|\;\right\|}_{2}$ is the Euclidean norm operation and *r_i_*(*t*) is the coordinate of atom *i* at time *t*. By statistical average, the displacement per atom $\bar{d}$ is computed herein as the root mean squared displacement (RMSD), as described in Equation (3):

(3) $$\bar{d}=\sqrt[{2}]{\langle {d_{i}}^{2}\rangle },$$

wherein < > is an average operation. Based on the mean squared displacement (MSD) at a long timescale, the average atom diffusion coefficient *D* is then computed according to the Einstein equation formulated in Equation (4) [33]:

(4) $$D=\underset{t\to \infty }{\lim}\frac{MSD}{6t}=\underset{t\to \infty }{\lim}\frac{{\bar{d}}^{2}}{6t}.$$

Next, in order to identify the factors affecting atom motions, the atom potential energy is computed by summing up all its interactions with other atoms, and the atom Voronoi volume is computed by the Voronoi tessellation method to characterize the atom free volume [37,51]. The atom stress Pi that characterizes the local stress imposed on each atom is computed by Equation (5) [52]:

(5) $$P_{i}=\frac{m_{i}v_{i}^{2}+{\vec{r}}_{i}\cdot {\vec{F}}_{i}}{3V_{i}},$$

wherein *V_i_*, *m_i_*, *v_i_*, and ${\vec{r}}_{i}$ are the volume, mass, velocity, and position of atom *i*, respectively, and ${\vec{F}}_{i}$ is the resultant force applied on atom *i* by all the other atoms in the system. *P_i_* consists of three normal stress components {$\sigma_{i}^{x}$, $\sigma_{i}^{y}$, $\sigma_{i}^{z}$} along the x, y, and z axis, and three shear stress components {$\tau_{i}^{xy}$, $\tau_{i}^{yz}$, $\tau_{i}^{xz}$} in the xy, yz, and xz plane, respectively. Note that although the graft PP systems as a whole is at zero pressure, some atoms are under compression while others are under tension, so that they mutually compensate each other. By convention, a positive normal stress represents a state of tension, whereas a negative one represents a state of compression.

The atom normal stress $\sigma_{i}$ is computed herein by averaging the three normal stress components, as described in Equation (6):

(6) $$\sigma_{i}=\frac{\sigma_{i}^{x}+\sigma_{i}^{y}+\sigma_{i}^{z}}{3},$$

while the atom shear stress $\tau_{i}$ is computed using the von Mises definition formulated in Equation (7) [35]:

(7) $$\tau_{i}=\frac{\sqrt{{{(\tau }_{i}^{xy})}^{2}+{{(\tau }_{i}^{yz})}^{2}+{{(\tau }_{i}^{xz})}^{2}}}{3}.$$

Based on the atom stress computation, the normal and shear stress per atom $\bar{\sigma }$ and $\bar{\tau }$ are computed by statistical average using $\bar{\sigma }=\langle \left|\sigma_{i}\right|\rangle$ and $\bar{\tau }=\langle \tau_{i}\rangle$, respectively, wherein the absolute value of normal stress $\left|\sigma_{i}\right|$ is used to characterize the magnitude of compression and tension stress.

## 3. Results and Discussion

### 3.1. One-Shot Screening of Graft Effect on Chain Mobility

**Figure 3:**
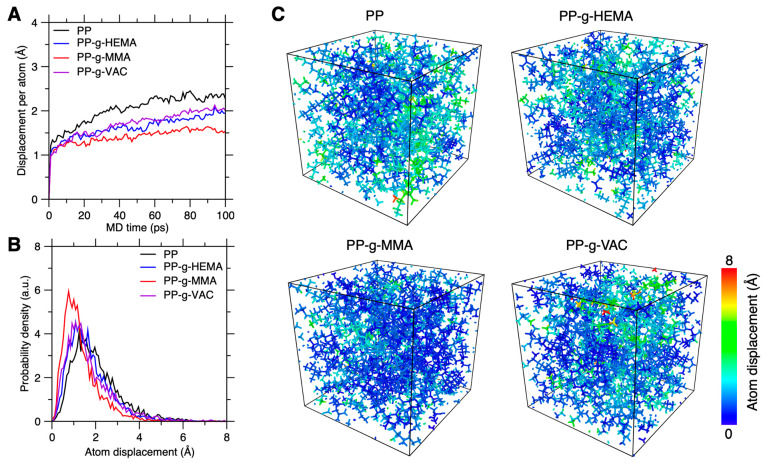
Graft PP chain mobility characterized by atom displacement. (**A**) Evolution of displacement per atom over time in graft PP systems under NPT ensemble. (**B**) Their probability density distribution of atom displacement after a time duration of 100 ps. (**C**) Color coding of the graft PP configurations based on their atom displacement after 100 ps.

We first investigate the graft effect on PP chain mobility based on atom motion trajectories of one equilibrated configuration. The simulation details are provided in Section 2.2. This “one-shot” screening adopts one representative configuration for chain dynamics analysis and is suitable for fast screening of numerous side-chain types at a minimal computation cost. Figure 3A shows the evolution of displacement per atom with time in the pristine and graft PP systems. Due to their structural similarity, the three graft PP systems exhibit an approximately non-evident displacement distinction less than 0.5 Å. We nevertheless find that all the side-chain types tend to stabilize PP chains with a possible chain mobility rank of PP-g-MMA > PP-g-VAC ≈ PP-g-HEMA > PP.

We then examine the atom-level picture of graft PP chain dynamics. Figure 3B provides their atom displacement distribution after 100 ps. Indeed, we find that the PP-g-MMA shows the narrowest distribution of atom displacement with a peak centered around 1 Å, while the pristine PP exhibits the widest distribution with the peak shifted to 2 Å, and the similar distributions of PP-g-VAC and PP-g-HEMA lie in between them, with the PP-g-VAC peak position slightly smaller than that of PP-g-HEMA. These results confirm the promising stabilization effect of these side chains on PP chain dynamics, especially for the PP-g-MMA system.

Figure 3C further shows their final configurations color-coded by atom displacement. Indeed, by grafting side chains onto PP backbones, we find that the backbones become stiffer to freeze chain dynamics in terms of both the atom displacement magnitude and the low-displacement area scope. This stabilization phenomenon is likely attributed to the increased steric hindrance effects and intermolecular interactions derived from these side chains [4]. Moreover, we find that the high-mobility regions largely arise from (i) the segments near chain-end regions and (ii) the segments in loosely packed regions, which are prone to activate with less steric hindrance effect. Overall, the one-shot screening presents a computationally efficient yet precise and reliable method to establish a fast evaluation of graft effect on chain mobility.

### 3.2. Identification of Atomic Features Responsible for Graft Chain Mobility

We now take a closer atom-level inspection into the possible dominant factors governing atom displacement, aiming to identify certain atomic features (if any) responsible for graft chain mobility. This feature identification constitutes an active research subfield to promote atomistic and molecular system design, guided by pre-identified structural or non-dynamical features [33,38,40]; however, it remains very challenging to predict complex atom dynamics without deliberate feature construction strategies [40]. In that regard, we examine herein the correlation of atom displacement with its atom potential energy (see Section 3.2.1), atom Voronoi volume (see Section 3.2.2), and atom stress (see Section 3.2.3), which characterizes, respectively, the energy-favored stability, the free-volume-favored stability, and the stress-favored stability that likely affect the atom mobility and chain dynamics.

#### 3.2.1. Correlation Between Atomic Energy and Chain Mobility

**Figure 4:**
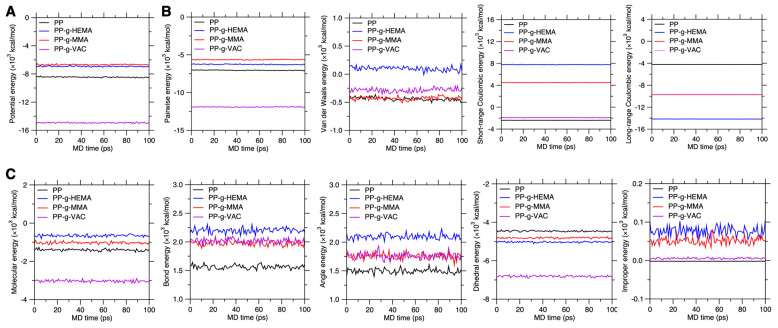
Potential energy of the graft PP systems. (**A**) Potential energy of graft PP systems recorded for a time duration of 100 ps. The energy is a summation of pairwise energy (**B**) and molecular energy (**C**). (**B**) Comparison of pairwise energy in these graft systems. The energy consists of van der Waals energy and short- and long-range Coulombic energy. (**C**) Comparison of their molecular energy, which consists of bond, angle, dihedral, and improper energy terms (see Equation (1)).

Firstly, we investigate the graft effect on potential energy of the equilibrated configuration. By summing up all interatomic interactions, the potential energy is an indication of whether the graft PP system is packed by energy-favored chain conformation. Figure 4A shows the potential energy of graft PP systems recorded for 100 ps, and the corresponding pairwise and molecular energy components are provided in Figure 4B,C. Notably, compared to the pristine PP system, the PP-g-VAC system exhibits a much lower potential energy, while the PP-g-MMA shows enhanced potential energy, similar to that of PP-g-HEMA. These results suggest that the PP-g-VAC chain conformation is energy-favored, while the other two graft conformations remain energy-stable but are less energy-favored. Consequently, this energy-favored stability is likely to suppress chain mobility to some extent, so as to lower atom displacement in PP-g-VAC. However, it should be pointed out that a higher-energy state does not necessarily promote chain mobility, similar to the PP-g-MMA configuration, which is likely ascribed to its deeply energy-stable state [20].

Then, we compare the pairwise and molecular energy components that cause the energy-favored stability (see Figure 4B,C). It is notable that the pairwise Coulombic energy term plays a dominant role in determining the magnitude of system potential energy (see Figure 4B), which could in turn affect the molecular energy components, with PP-g-VAC and PP-g-HEMA exhibiting the lowest and highest molecular energy, respectively (see Figure 4C). By comparing the bond, angle, dihedral, and improper energy, we find that all side-chain types tend to decrease the dihedral energy but increase the bond, angle, and improper energy, with the highest increment from PP-g-HEMA, suggesting that the Coulombic interaction from side chains tends to distort the local chain conformation via the short-range bond, angle, and improper structures. This local distortion would possibly result in more stress-induced instability and, therefore, higher mobility of local chain segments. Moreover, the hydrogen bonding plays a critical role in stabilizing chain dynamics and lowering the system potential energy. We describe herein the various sorts of interactions by COMPASS forcefield, with the hydrogen bonding incorporated into the van der Waals energy and Coulombic energy. These interactions are mutually dependent and synergically determine the topography of potential energy landscape (PEL) and the resultant chain dynamics.

**Figure 5:**
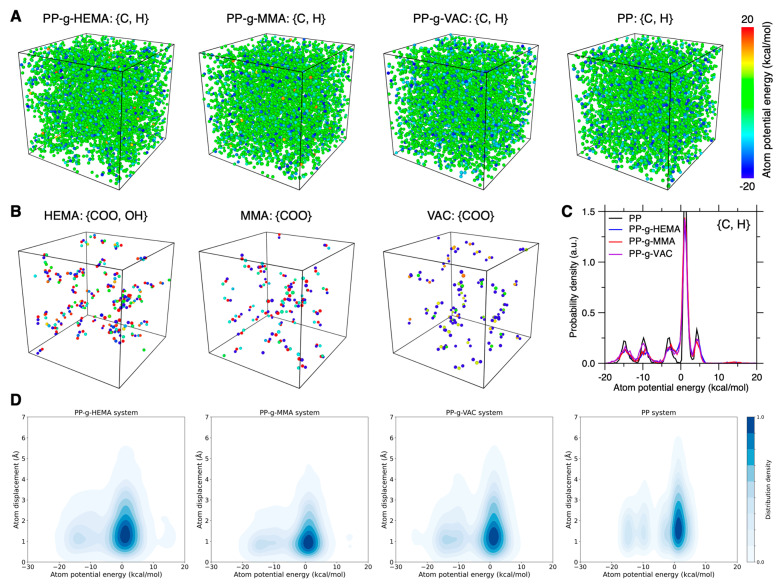
Atomic energy effect on graft PP chain mobility. (**A**) Color coding of the CH*_n_*_=0,1,2,3_ unit atoms in graft PP configurations based on their atom potential energy. (**B**) The corresponding color coding of O–C=O and O–H group atoms in side chains of these configurations. (**C**) Probability density distribution of atom potential energy in CH*_n_*_=0,1,2,3_ units of these configurations. (**D**) Probability density distribution as a function of the initial atom potential energy and its final atom displacement after 100 ps duration in these graft PP systems.

Further, we investigate the local distribution of interaction energy using atom potential energy, i.e., its overall interactions with its neighboring atoms. Figure 5A,B show the spatial distribution of atom potential energy for, respectively, the atoms in all CH*_n_*_=0,1,2,3_ units and the atoms in the side-chain O–C=O and O–H groups. And the probability density distribution of atom potential energy for CH*_n_*_=0,1,2,3_ units are provided in Figure 5C. Compared to the pristine PP configuration, we find that the graft PP backbone atoms show an approximately similar atom potential energy magnitude and distribution pattern, with only a few higher-energy carbon atoms in PP-g-HEMA and PP-g-MMA configurations (see Figure 5A). In contrast, the side chains of PP-g-HEMA and PP-g-MMA generally exhibit high-energy carbon atoms for the ester group and high-energy oxygen atoms for the hydroxyl group (see Figure 5B), thus resulting in the increase in system potential energy. Notably, despite their structural similarity, the ester group in the PP-g-VAC side chain exhibits significantly lower atom potential energy than that of PP-g-MMA, suggesting the significance of the ester group connection sequence in determining the energy-favored stability of side-chain conformation.

Based on these results, we now examine the correlation between atom displacement and atom potential energy. Figure 5D shows the probability density distribution as a function of the initial atom potential energy and its final atom displacement after 100 ps for the pristine and graft PP configurations. Indeed, we find that the higher-energy atoms have a tendency to exhibit larger atom displacement, but there remain many atoms at a high-energy but low-mobility state. Moreover, the similar distribution pattern of atom potential energy does not explain why the PP-g-MMA system exhibits the lowest atom displacement on average. As such, it is concluded that there is only a weak-to-modest correlation between atom displacement and atom potential energy, which implies that, despite some anticorrelation between energy level and stability, the energy level of an atom does not necessarily reflect its energy stability.

#### 3.2.2. Correlation Between Atomic Voronoi Volume and Chain Mobility

**Figure 6:**
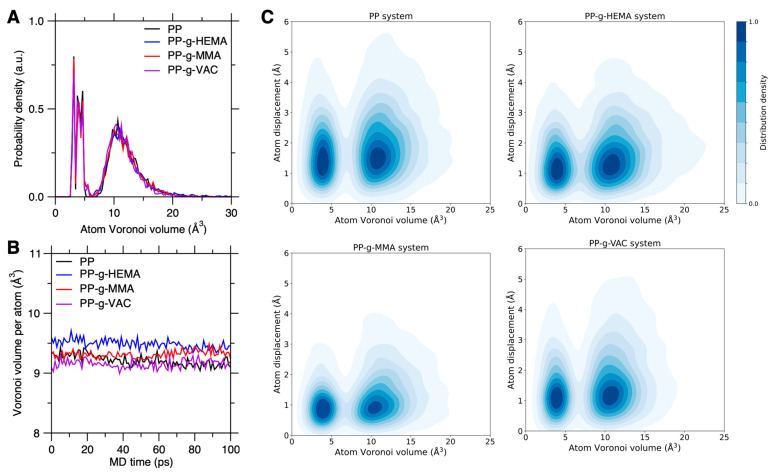
Voronoi volume effect on graft PP chain mobility. (**A**) Probability density distribution of atom Voronoi volume in graft PP configurations. (**B**) Voronoi volume per atom recorded for a time duration of 100 ps in these graft PP systems. (**C**) Probability density distribution as a function of the initial atom Voronoi volume and its final atom displacement after 100 ps duration in these systems.

Next, we investigate the structural origin of graft chain mobility by examining the correlation between atom displacement and atom Voronoi volume. By characterizing the free volume of each atom, the atom Voronoi volume is an indication of steric hindrance effect in graft PP systems. Figure 6A shows the probability density distribution of atom Voronoi volume in pristine and graft PP systems, wherein the distributions are largely overlapped, with a similar atom Voronoi volume in each system. By statistical averaging, Figure 6B shows the Voronoi volume per atom recorded for 100 ps in these systems. We find that all the configurations show an approximately similar average Voronoi volume ranging from 9.0 to 9.5 Å^3^, with PP-g-HEMA and PP-g-VAC exhibiting the largest and smallest Voronoi volume, respectively. This result suggests the apparent steric hindrance effect in PP-g-VAC to constrain chain segment motions.

To unveil the Voronoi volume effect on chain dynamics, Figure 6C shows the probability density distribution as a function of the initial atom Voronoi volume and its final atom displacement after 100 ps for graft PP systems. Indeed, ascribed to the reduced steric effect, a larger Voronoi volume generally leads to an increased tendency of atom displacement, but there remain many atoms at a high-free-volume but low-mobility state, and vice versa. Moreover, similar to the atomic energy (see Figure 5C), the similar distribution pattern of the atom Voronoi volume does not explain the low-mobility tendency in PP-g-MMA. Therefore, despite some weak-to-modest correlation between atom Voronoi volume and its atom displacement, the graft chain dynamics must be synergically governed by both the structural and non-structural features (e.g., local stress) at the atomistic scale.

#### 3.2.3. Correlation Between Atomic Stress and Chain Mobility

**Figure 7:**
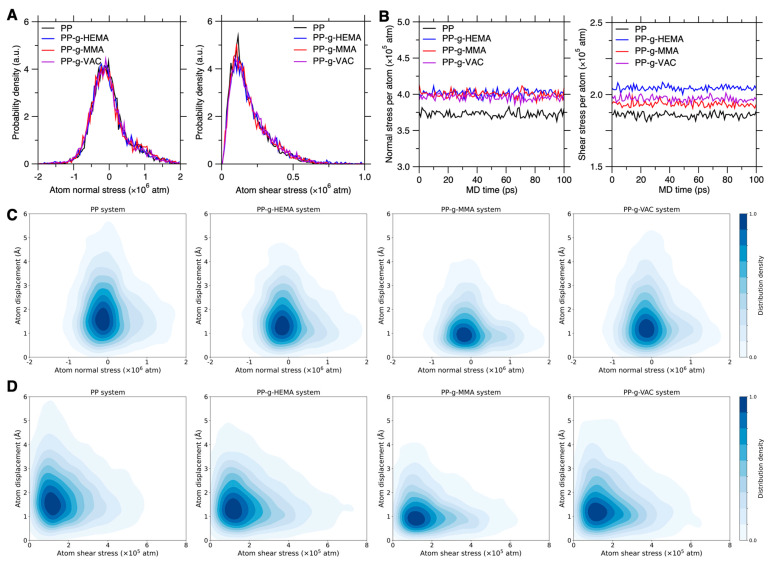
Atomic stress effect on graft PP chain mobility. (**A**) Probability density distribution of atom normal and shear stress in graft PP configurations. (**B**) Normal and shear stress per atom recorded for a time duration of 100 ps in these graft PP systems. (**C**) Probability density distribution as a function of the initial atom normal stress and its finial atom displacement after 100 ps duration in these systems. (**D**) Its counterpart distribution as a function of atom shear stress and its final displacement.

Herein, we examine the correlation between atom displacement and atom stress, including both atom normal stress and atom shear stress (see Equations (6) and (7) in Section 2.3), in order to unveil the local stress effect on graft chain dynamics. By characterizing the local stress experienced by each atom, the atom stress is an indication of the local mechanical stability in graft PP systems. Figure 7A shows the probability density distribution of atom normal and shear stress in pristine and graft PP configurations, wherein the normal stress distribution is nearly symmetric, with a peak centered around zero to maintain the zero-pressure state. Note that all configurations have been fully relaxed to release the internal stress to an inevitable and intrinsic stress state. We find that all the systems show approximately similar atom stress distributions that are nearly overlapped with each other. By statistical averaging, Figure 7B shows their normal and shear stress per atom recorded for 100 ps. Notably, compared to the pristine PP system, all the side-chain types increase both the normal and shear stress per atom, with the normal stress increased in a similar trend, while the PP-g-HEMA atoms are subjected to the largest shear stress on average, suggesting the potential low mechanical stability and high-mobility tendency of PP-g-HEMA.

Figure 7C and Figure 7D further provides their probability density distribution as a function of, respectively, the initial atom normal and shear stress and its final atom displacement after 100 ps. Indeed, higher-stress atoms exhibit a modestly increased tendency of atom displacement, but in contrast, it is the zero- or low-stress atoms that constitute the majority of high-mobility atoms. Moreover, similar to atomic energy and volume (see Figure 5C and Figure 6C), the similar distribution pattern of atom stress does not explain the low-mobility tendency of PP-g-MMA. These results suggest that the initial atom stress is not mainly responsible for the atom displacement discrepancy in the pristine and graft PP systems. And rather than the static state of local stress and its mechanical stability, the dynamical evolution of local stress-induced mechanical stability plays a direct role in controlling graft chain dynamics.

Overall, based on all the correlation analysis, i.e., correlating atom displacement to its atom potential energy, atom Voronoi volume, and atom normal and shear stress, it is concluded that the graft chain dynamics are not governed by these static structural and interaction features in a simple intuitive manner. More synergic effects of various structural and interaction features should be taken into account, so as to identify some nonintuitive static features responsible for predicting complex chain dynamics, such as the atom softness approach for atom dynamics prediction [38]. Essentially, from the PEL viewpoint [25], the complex chain dynamics are fundamentally governed by the PEL topography, which is constituted with the pair description of structure and interaction in the PEL space (see Section 3.4). The nonintuitive, synergic static feature (if any) should somehow describe the local PEL topography, so as to predict the complex chain dynamics solely from static structure and interaction features [31,33,38]. More investigation efforts are needed in that direction. In the following sections, rather than using static features to predict graft chain mobility, we directly evaluate the graft chain mobility and polymer thermal stability based on atom motion trajectories of several parallel configurations sampled across PEL, which in turn assists in depicting the local or more global PEL topography that governs graft chain dynamics.

### 3.3. Sampling-Average Screening of Graft Chain Mobility

**Figure 8:**
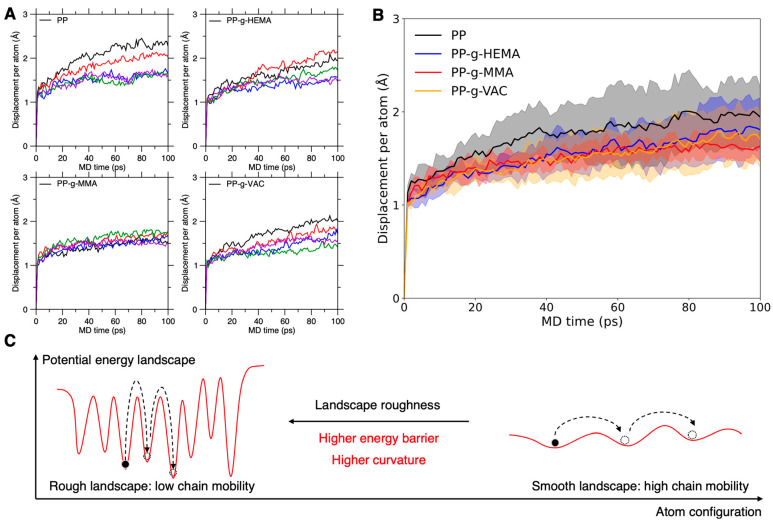
Initial configuration effect on graft PP chain mobility. (**A**) Evolution of displacement per atom over time in graft PP systems under NPT ensemble using distinct initial configurations. The line color coding is based on the same sampling protocol of initial configurations, which are extracted from a relaxation trajectory at elevated temperature under NVT ensemble. (**B**) Statistical average of displacement per atom over time in these graft PP systems. The shadow region indicates the atom displacement span under distinct initial configurations. The upper and lower shadow boundary are set as the observed maximum and minimum displacement, while averaging the two extreme boundaries offers an approximate estimation of the average displacement per atom. (**C**) Illustration of the potential energy landscape’s roughness governing chain mobility. Higher energy barrier and curvature lead to rougher energy landscape, thus suppressing atom mobility and promoting chain stability.

Now we investigate the initial configuration effect on graft chain mobility using atom motion trajectories of several parallel configurations, which are sampled at an elevated temperature and re-equilibrated to 300 K for atom trajectory recording (see Section 2.2). Note that despite the non-evident correlation between graft chain dynamics and their initial structure features, such as the atom Voronoi volume (see Section 3.2.2), the initial configuration plays a dominant role in determining chain dynamics, considering the intrinsic structure–dynamics relationship [31,40]. Figure 8A shows the evolution of displacement per atom over time in the pristine and graft PP systems using distinct initial configurations. As expected, we find that the different initial configurations lead to a close but distinct displacement per atom within a variation of 1 Å, demonstrating a strong correlation between graft chain dynamics and its initial static structure.

Taking the initial configuration effect into consideration, we further evaluate the graft PP systems’ chain segment mobility and their thermal stability in a more reliable manner by the initial-configuration-sampling-based statistical average, termed “sampling-average” screening. Unlike the one-shot screening using only one initial configuration (see Section 3.1), this “sampling-average” screening adopts several samplings of representative initial configurations for statistical average analysis of chain dynamics variation across different samplings. Note that a proper sampling strategy is needed to ensure the correctness of statistical results (see Section 3.6). This analysis significantly enhances the screening reliability and is a normal practice in various simulation studies [53], with the computation cost scaling up linearly with the number of samplings, suitable for refined screening of competitive side-chain types at a minimal computation cost.

By conducting the sampling-average screening, Figure 8B shows the statistical average of the distributed displacement per atom over time in these graft PP systems, wherein the upper and lower distribution boundary are set as the observed maximum and minimum displacement, and averaging the two extreme boundaries offers an approximate estimation of the average displacement per atom. We find that the mean ± standard deviation calculation offers fluctuation results similar to the max–min boundary approach and does not affect the conclusions in this work (see Supplementary Materials). Indeed, the pristine PP system exhibits the highest average displacement per atom, posteriorly validating the graft effect on stabilizing the chain dynamics. But in accordance with their structural similarity, the graft PP systems shows an approximately similar average displacement per atom, and the distributed displacement per atom of these systems are largely overlapped with each other. Notably, the PP-g-MMA and PP systems exhibit the smallest and largest distribution variation range, respectively, which confirms the low-mobility tendency of PP-g-MMA. Moreover, featured by a medium variation, the PP-g-VAC system exhibits the lowest distribution boundary across all configuration samplings, which presents an indication of its low-mobility tendency.

Based on these analyses, we adopt three statistical metrics significant to evaluate graft chain mobility, including the average, variation range, and minimum boundary in the distributed displacement per atom, wherein a perfectly stabilized polymer system should exhibit the combined metrics of low average, small variation range, and low minimum boundary. Relying on the three metrics of graft chain mobility, the sampling-average screening yields (i) an “average” metric rank of PP > PP-g-HEMA ≈ PP-g-VAC ≥ PP-g-MMA, (ii) a “variation-range” metric rank of PP > PP-g-HEMA ≈ PP-g-VAC > PP-g-MMA, and (iii) a “minimum-boundary” metric rank of PP ≥ PP-g-HEMA ≈ PP-g-MMA > PP-g-VAC (see Figure 8B). Altogether, these results would establish temporarily an overall chain mobility rank of PP > PP-g-HEMA ≥ PP-g-VAC ≈ PP-g-MMA, wherein PP-g-MMA and PP-g-VAC are two promising candidates to stabilize PP chain dynamics, slightly better than PP-g-HEMA. Nevertheless, it remains challenging to differentiate their overall chain mobility based on the largely overlapped distributions shown in Figure 8B, and more refined screening strategies are needed to distinguish their thermal stability (see Section 3.4 and Section 3.5).

### 3.4. Energy Landscape Interpretation of Graft Chain Mobility

In order to rationalize the graft-effect screening at a finer precision resolution, we now delve into the potential energy landscape (PEL) that governs the graft chain dynamics. Essentially, all atom motions are guided by their local PEL topography, as illustrated in Figure 8C, wherein a rough landscape reduces chain mobility, while a smooth landscape promotes atom motions [21,25]. In other words, higher PEL “roughness” would lead to a lower atom displacement, which in turn provides an indication of PEL roughness. Note that the roughness arises from a synergic effect of the energy barrier and its basin curvature [54], wherein both the higher energy barrier and basin curvature would lead to higher PEL roughness, thus reducing the graft chain mobility (see Figure 8C). By tuning the complex interatomic interactions, e.g., grafting side chains, the energy barrier and basin curvature are likely modified dramatically or modestly to wrinkle (or flatten) the PEL, resulting in increased (or decreased) roughness and chain stability.

According to the distributed results of displacement per atom in Figure 8B, we then estimate the PEL roughness distributions of these systems following their displacement variation pattern. The pristine PP system exhibits the widest roughness distribution, with an average roughness slightly smaller than the graft PP systems (see Figure 8B), wherein PP-g-MMA shows the narrowest roughness distribution, and PP-g-VAC exhibits some local PEL regions with the largest maximum roughness. These results characterize the representative PEL roughness distribution and somehow depict the local or more global PEL topography, providing a fundamental interpretation of the diversity in graft chain mobility from different side-chain types or different initial configurations. Nevertheless, it remains challenging to differentiate graft chain mobility from the largely overlapped roughness distribution. And an approach to quantify the overall roughness is needed to yield an overall rank of graft chain mobility and thermal stability thereof. In that regard, the Arrhenius-activation-based formulation of atom diffusion at various temperatures provides an opportunity to quantify the overall roughness via estimating the overall energy barrier of atom diffusion [21] (see Section 3.5).

### 3.5. Arrhenius-Dependance Screening of Graft Chain Mobility

In order to properly quantify the PEL roughness via energy barrier computation, we introduce herein the Arrhenius-activation-based formulation of atom diffusion, as described in Equation (8) [21,33]:

(8) $$D=D_{0}\exp\left(\frac{E_{A}}{k_{B}T}\right),$$

wherein the atom diffusion coefficient *D* is a function of temperature *T* and is computed by the Einstein equation (see Equation (4)), *D*_0_ denotes the pre-exponential factor, *k_B_* is the Boltzmann constant, and *E_A_* is the average activation energy for atom migration and provides a quantification of the overall PEL roughness. Note that *E_A_* can also be computed directly for each atom via local PEL saddle point search algorithms [54], but this comes with a significant computation cost for large molecular systems, and more investigation efforts are needed in that direction. Herein, we conduct atom diffusion simulations of the equilibrated systems at an extended timescale up to 1 ns and elevated temperatures from 300 K to 500 K with an increment of 50 K under the NVT ensemble (see Section 2.2), so as to compute the atom diffusion coefficient using Equation (4) and estimate the energy barrier and PEL roughness by Equation (8).

**Figure 9:**
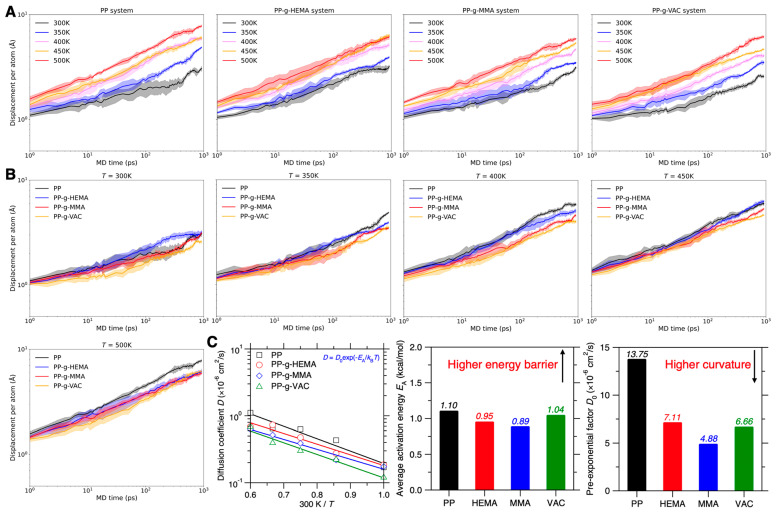
Arrhenius dependance of graft PP chain mobility at elevated temperatures. (**A**) Evolution of displacement per atom over time up to 1 ns in graft PP systems under NVT ensemble at elevated temperatures from 300 to 500 K with an increment of 50 K. The shadow region quantifies the span of displacement per atom under distinct initial configurations, wherein, for simplicity, different start configurations in the first 10% trajectory itself are set as the initial configurations. (**B**) Counterpart plot of displacement per atom over time in these graft PP systems at the same temperature, ranging from 300 K to 500 K in a row. (**C**) Atom diffusion coefficient *D*(*T*) as a function of the inverse temperature 300/*T*. The fitting lines indicate Arrhenius-type activation process following Equation (8), with the R^2^ score equal to 0.9414, 0.9566, 0.9677, and 0.9710 for PP, PP-g-HEMA, PP-g-MMA, and PP-g-VAC, respectively. Based on the fitting, the average activation energy *E*_A_ (middle panel) and the pre-exponential factor *D*_0_ (right panel) are provided to characterize energy landscape roughness of these graft PP systems, that is, higher *E*_A_ and lower *D*_0_ signify rougher landscape.

Relying on the Arrhenius-type activation process, the graft chain mobility screening is herein termed as “Arrhenius-dependance” screening. Figure 9A shows the evolution of displacement per atom over time at various temperatures in the pristine and graft PP systems, with the displacement statistically averaged over distinct initial configurations. Note that the extended-timescale simulation required by Equation (4) could require a surging computation cost (see Section 3.6) and, for simplicity, different starting configurations in the first 10% trajectory itself are set as the initial configurations for the statistical average, wherein different sampling strategies could potentially affect the distributed displacement (see Section 3.6), but does not hinder the Arrhenius-equation-based overall roughness estimation. By increasing the temperature, all the systems show a larger displacement per atom, with the pristine PP system exhibiting the largest increment of ~7 Å, suggesting its high-mobility tendency and thermal instability.

To further compare the graft effect on chain mobility, Figure 9B provides the evolution of displacement per atom over time across graft PP systems at the same temperature, ranging from 300 K to 500 K. At a low temperature of 300 K, the displacement per atom after 1 ns shows a non-evident discrepancy, with PP-g-VAC and PP-g-HEMA exhibiting the smallest and largest displacement, respectively. Note that under the NVT ensemble, the displacement trend herein could be slightly different from the previous sections using the NPT ensemble (see Figure 3 and Figure 8), so as to eliminate the volume expansion effect at an elevated temperature and characterize the local roughness of the present initial configuration. To enhance the screening precision, more distinct initial configurations are expected for the statistical average to entirely encompass the various displacement trend, which would broaden and deteriorate the precision resolution in displacement comparison at each temperature, but the temperature-dependent displacement trends should generally remain unaltered as a physics principle in Equation (8) for reliable PEL roughness estimation. Indeed, by slightly increasing the temperature to 350 K, the displacement trends become in excellent agreement with the trends shown in previous sections. Moreover, by extending the timescale up to 1 ns, the precision resolution becomes narrowed down for a more distinctive displacement comparison, which is likely ascribed to the reduced roughness difference at a more global PEL inspection scope (see Section 3.6).

We then compare the displacement per atom after 1 ns in these graft systems at various temperatures T by computing their atom diffusion coefficient D(T) (see Equation (4)), and the results are shown in Figure 9C. Note that the D value herein is underestimated to some extent using the sub-diffusive regime displacement at 1 ns, but it nevertheless reflects the D evolution trend in an approximate fashion. Notably, by sequentially increasing temperature from 300 K to 500 K, the pristine PP system generally exhibits the highest D value in terms of the overall tendency, while the PP-g-VAC system shows the smallest D value, despite some deviation at certain temperatures, which is likely rooted in the temperature-dependent PEL roughness resolution (see Section 3.7). Based on the temperature-dependent D trends, we apply the Equation (8) to fit the trend and establish a finalized chain mobility rank of PP-g-VAC ≥ PP-g-MMA > PP-g-HEMA > PP (see Figure 9C). Note that by relying on Arrhenius-dependance screening, this ranking result is close to that of one-shot or sampling-average screening and exhibits enhanced reliability. Importantly, by extending the timescale and elevating the temperature, this approach enables differentiating chain dynamics of the two competitive promising systems, PP-g-VAC and PP-g-MMA, which are challenging to distinguish in terms of the stabilization effect of their side chains.

Moreover, according to the Arrhenius-type fitting trend by Equation (8), we further estimate the graft PP systems’ PEL roughness and their PEL topography characteristics via the two fitting parameters EA and D0. The results of EA and D0 are shown in Figure 9C, wherein the average activation energy EA characterizes the overall energy barrier, while D0 is the diffusion coefficient at infinite temperature [33]. By eliminating all energy barrier influences on atom motions, D0 essentially characterizes the overall curvatures of energy basins, and more curved basins result in a more wrinkled PEL topography, thus lowering the atom displacements [20,54] (see Figure 8C). It is interesting that all graft PP systems show close but smaller EA values than the pristine PP system (see Figure 9C), while their D0 values exhibit merely half of the PP system’s D0 value, with PP-g-MMA exhibiting both the lowest EA and D0 value. Considering their overall mobility rank, these results suggest that the graft PP systems enhance the PEL roughness through a counterbalance between possibly lowering the overall energy barrier but extensively wrinkling the landscape by increasing the overall curvature of energy basins. Note that this inference could be affected by the fitting precision, and more deliberate investigations are needed in that direction, e.g., computing the atom energy barrier via PEL saddle point search algorithms [28,54].

### 3.6. Thermal Stability Comparison by Three-Stage Virtual Screening

**Figure 10:**
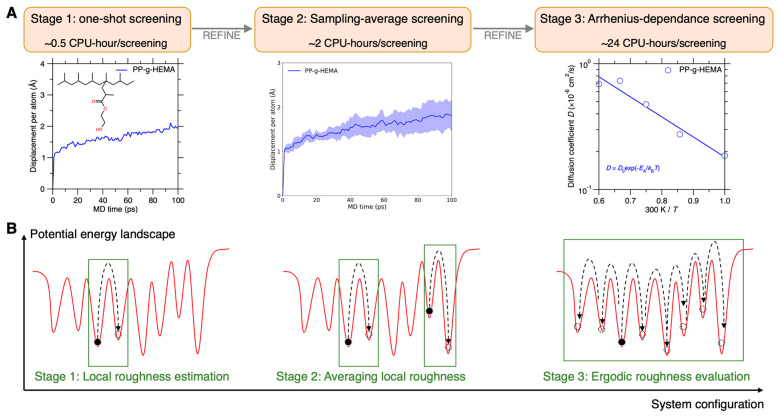
Three-stage virtual screening of graft PP chain mobility. (**A**) Workflow of the three-stage screening, including one-shot, sampling-average, and Arrhenius-dependance screening, with a computation cost of ~0.5, ~2, and ~24 CPU-hours, respectively. These costs can also be estimated based on a reference test of ~20mins/5000 atoms/1M steps at 56 cores/CPU-3F at National Supercomputing Center (NSCC) clusters. (**B**) Energy landscape interpretation of the three-stage screening. The one-shot screening in stage 1 is essentially estimating one local roughness, which is less accurate but low computation cost in evaluating graft PP chain stability from numerous side-chain types, while stage 2 and 3 sequentially broaden the estimation region of energy landscape, therefore enhancing the estimation accuracy with more computation cost, suitable for screening refinement of competitive side chains.

Based on all these aforementioned screening strategies, we establish herein the three-stage virtual screening pipeline to differentiate the graft effect on chain mobility, including the stage of one-shot, sampling-average, and Arrhenius-dependence screening, as illustrated in Figure 10A. The three-stage screening rationalizes chain dynamics evaluation in stepwise higher precision while minimizing the computation cost, with a screening speed of ~0.5, ~2, and ~24 CPU hours per screening for stage 1, 2, and 3, respectively. Notably, by extending timescale at elevated temperatures, the stage 3 screening exhibits a finer precision resolution to distinguish chain mobility from similar competitive side-chain types. From the PEL viewpoint [20,27,41], this enhancement of precision resolution is attributed to the extended inspection scope of PEL roughness, i.e., from (i) estimating one local roughness in stage 1, (ii) averaging multiple local roughness in stage 2, to (iii) quantifying more ergodic roughness in stage 3, as illustrated in Figure 10B. Herein, sampling from each PEL position, the local roughness exhibits a wide distribution, while under an extended timescale and elevated temperature, the more ergodic roughness inspection significantly narrows down the roughness fluctuation at more global PEL scope.

Despite the wider roughness distribution at the local PEL scope, the one-shot screening in stage 1 is valuable for fast screening of numerous side-chain types at a minimal computation cost, assuming that the “one-shot” configuration is generally representative of numerous graft PP chain conformations. In the same spirit, the local PEL roughness of one configuration is assumed to be representative of the roughness across the global PEL space. Although this assumption is not rigorously correct, we find that the sampling representativeness generally holds true across the explored PEL space, based on the evidences of (i) the narrowly distributed displacement using distinct initial configurations (see Figure 8B) and (ii) the consistent evolution trend of temperature-dependent chain mobility in these graft PP systems (see Figure 9C), which implies that the PEL roughness sampling at one temperature is also representative of roughness samplings at different temperatures (see Section 3.7).

Note that in accordance with the sampling representativeness in stage 1, a proper sampling strategy is needed to ensure the correctness of all the three stage screenings. Herein, we adopt the typical high-temperature sampling strategy, along with a proper thermal history in terms of timescale and temperature (see Section 2.2), to obtain one or several equilibrated configurations that are considered representative [39,55,56]. And herein, we conduct five configuration samplings for statistical average in the stage 2 and 3 screening. Since the number of samplings remains small to minimize the computation cost of independent parallel simulations, this work adopts the minimum and maximum value to compute the statistical average (see Figure 8 and Figure 9), so as to put more emphasis on individual sampling representativeness. Ideally, if considerably more samplings were used for the statistical average, it would be a standard practice to compute the mean and standard deviation to fully respect the statistical nature of numerous samplings. This might broaden the statistical distribution to some extent (see Figure 8B and Figure 9A), thus lowering the screening precision resolution in graft chain mobility at each temperature; however, this does not modify the overall fitting trend of Arrhenius-type activation process in the stage 3 screening (see Figure 9C), enabling the ergodic-state differentiation of graft chain mobility in finest precision resolution—comparing to other ergodic roughness metrics like glass transition temperature (see Section 3.7).

### 3.7. Precision Resolution of Ergodic Roughness Metrics

**Figure 11:**
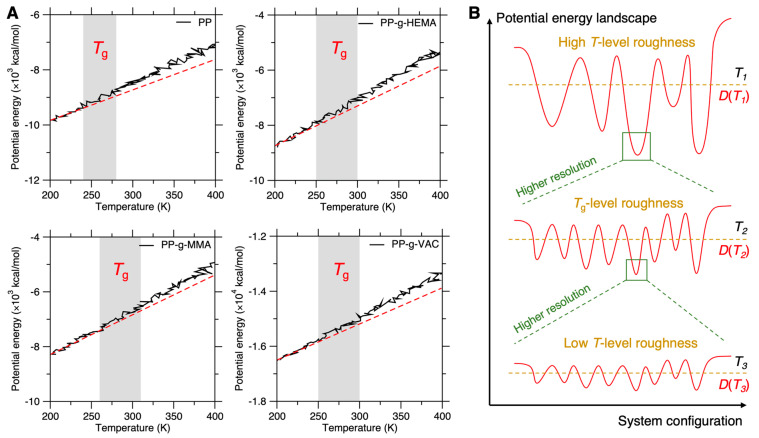
Precision resolution of ergodic roughness metric using glass transition temperature. (**A**) Evolution of potential energy with temperature for graft PP systems during a cooling process from 400 K to 200 K in 100 ps under NVT ensemble. The red dash is a linear fit of the low-temperature region from 200 K to 225K. The shadow region starts to deviate from the linear fit and indicates the range of glass transition temperature *T*_g_. (**B**) Illustration of PEL roughness resolution at different temperatures. Lower temperature (and timescale) leads to finer resolution of PEL roughness at less ergodic state. Diffusion coefficient *D*(*T*) characterizes the roughness at each temperature *T*, while *T*_g_ characterizes the *T*_g_-level roughness.

Finally, we evaluate the precision resolution of ergodic roughness estimation in the stage 3 screening, which adopts the Arrhenius-type activation trend fitting by Equation (8). We compare it to the precision resolution of other ergodic roughness metrics like glass transition temperature *T*_g_, which is a broadly used metric to evaluate chain segment mobility and polymer thermal stability through ergodic roughness inspection across the global PEL space during the melt-quenching process [32,39,57]. Figure 11A shows the evolution of potential energy with temperature for the pristine and graft PP systems during a cooling process from 400 K to 200 K under the NVT ensemble. The simulation details are provided in Section 2.2. We find that the glass transition region, which deviates from a linear fit of the low-temperature region (see Figure 11A), can vary in a wide range and largely depend on the linear fit, so that the *T*_g_ metric is difficult to determine precisely within a wide variation range of ~50 °C, that is, a deficiently low precision resolution for virtual screening of competitive side-chain types. Indeed, we find that the *T*_g_ variation range of pristine and graft PP systems are largely overlapped, with the pristine PP exhibiting an average *T*_g_ of ~260 °C, consistent with experimental results, while the average *T*_g_ of graft PP systems are shifting to a higher temperature with a similar trend, with PP-g-MMA exhibiting the highest average *T*_g_, suggesting the stabilization effect of its side chains. However, the deficient precision resolution of *T*_g_ makes it incapable of differentiating all the competitive graft chain dynamics. Although its precision resolution could evolve better via a statistical average and deliberate sampling strategy, such as a slower cooling rate for a more well-defined glass transition region, the linear fitting approach for *T*_g_ determination remains a high uncertainty to lower the precision resolution.

Unlike the *T*_g_ metric, the Arrhenius-type activation trend fitting presents a significantly finer precision resolution, in terms of both the measurement resolution and PEL roughness resolution. Firstly, regarding the measurement resolution, although the statistical average might yield a modest distribution of atom diffusion coefficient D(T) for each system at each temperature T, the distribution width could be largely narrowed down by extending the timescale to more global inspection scope of PEL roughness. And more importantly, by elevating the temperature, the Arrhenius-type activation trend of D(T) in the statistical average would remain unaltered as a physics principle for each system. This trend line fitted by Equation (8) is in the finest measurement resolution and is then applied to differentiate the overall PEL roughness of graft PP systems in a preset temperature range (see Figure 9C). Moreover, the D(T) distribution itself at each temperature T further assists the local PEL roughness comparison at the temperature, despite a possible distribution overlap of D(T) across graft PP systems. As such, this complementary approach provides not only the overall chain mobility evaluation, but also a detailed evaluation of chain mobility distribution at a certain preset temperature, wherein the distribution may deviate from the overall chain mobility evaluation but is more reliable and applicable to this temperature (see Figure 9C), ascribed to the PEL roughness resolution (see below).

In addition to the finest measurement resolution, the Arrhenius-type activation trend also provides the finest resolution of PEL roughness at each temperature level, as illustrated in Figure 11B, wherein the PEL exhibits a hierarchical roughness resolution at different inspection scopes, with a more global scope of PEL roughness inspected at a higher temperature as well as a longer timescale. The PEL roughness at high and low temperatures are characterized by their D(T), respectively. We find that despite its hierarchical resolution, the PEL roughness at one temperature can generally serve as an indication of the roughness at different temperatures, considering the fact that the D(T) shows a nearly consistent ranking trend at each temperature (see Figure 9C). This representativeness of one-temperature roughness sampling echoes the effectiveness of the *T*_g_ metric in estimating ergodic roughness and polymer thermal stability, wherein the *T*_g_ metric is essentially solely indicative of the *T*_g_-level PEL roughness, with a higher *T*_g_ implying higher roughness at this temperature level, but can be indicative of the PEL roughness at different inspection scopes (see Figure 11B). However, we nevertheless find that at certain temperatures, the D(T) ranking trend might slightly deviate from the overall rank of the Arrhenius-type activation trend (see Figure 9C), suggesting that the local PEL roughness at this temperature level shows some nontrivial fluctuation, deviating from the overall ergodic roughness at a more global inspection scope. In turn, this deviation highlights the significance of local roughness estimation at a preset temperature using D(T) for validation in finer precision resolution. Overall, the hierarchical resolution of PEL roughness (i.e., characterized by D(T)) at each temperature synergically determines the ergodic roughness (i.e., characterized by the Arrhenius-type activation trend in Equation (8)) at the global PEL inspection scope, which governs the overall mobility of graft chains and their thermal stability, with each D(T) as a complementary validation to provide the finest precision resolution. While this work is a theoretical study, the computational results of the comparative trend offer an excellent agreement with the recent experimental finding of stabilizing graft PP chain dynamics by grafting polar groups [58], and the computational *T*_g_ results align well with experimental references [16,18], with an average *T*_g_ ≈ 260 K for pristine PP and shifting to a higher *T*_g_ by grafting polar groups, i.e., HEMA, MMA, and VAC herein, whose grafting effect on *T*_g_ and thermal stability remains lacking in experimental quantification but is efficiently accessible by the proposed computational protocol, ready to guide the experimental design of side-chain engineering.

## 4. Conclusions

To summarize, this work establishes a three-stage virtual pipeline, rationalized by the PEL theory in stepwise higher precision and computation cost, to sequentially refine the screening of the graft effect on chain mobility, which governs thermal stability, by taking the example of graft PP systems. This pipeline enables the differentiation of similar competitive side-chain types in the finest precision resolution. The stage 1 screening is essentially the local PEL roughness estimation of one equilibrated configuration, suitable for fast screening of numerous side-chain types to identify competitive graft chain dynamics. Then, by the statical average of configuration sampling across PEL, the stage 2 screening estimates the local roughness distribution for a more precise comparison of graft chain dynamics. Ascribed to their structural similarity, the graft PP systems exhibit a largely overlapped distribution of PEL roughness. By fitting the Arrhenius-type activation trend of the atom diffusion coefficient *D*(*T*) at different temperatures *T*, the stage 3 screening essentially estimates the ergodic roughness at a more global PEL inspection scope, so as to yield an overall rank of graft chain mobility and thermal stability. Overall, this work paves the way to evaluate graft chain dynamics and polymer thermal stability in silico, toward precise yet efficient side-chain engineering.

## Data Availability

All data needed to evaluate the conclusions of this study are present in the paper, and all relevant data are available from the corresponding author upon reasonable request.

## Supplementary Materials

The following supporting information can be downloaded at: https://www.mdpi.com/article/10.3390/polym17172298/s1, Figure S1: Mean ± standard deviation plot of atomic displacement over time, as a reference to the max–min boundary plot of Figure 8B in the main text.
