# Supplementary material for: Energy Landscape-Guided Virtual Screening of Side-Chain Engineering in Polymer Dynamics Design
Source: Polymers (Basel). 2025 Aug 25;17(17):2298. doi: 10.3390/polym17172298 (PMC12431039; doi:10.3390/polym17172298)
Supplement: Supplementary file 1 [file polymers-17-02298-s001.zip › polymers-3811571-supplementary.pdf]

Supplementary Materials to "Energy Landscape Guided Virtual Screening of Side Chain Engineering in Polymer Dynamics Design"

Han Liu [a,b] *, Sen Meng [a], Liantang Li [b]

[a] *Electric Power Research Institute, United Laboratory of Advanced Electrical Materials and Equipment Support Technology, China Southern Power Grid (CSG), Guangzhou 510663, China*
[b] *SOlids inFormaTics AI-Laboratory (SOFT-AI-Lab), College of Polymer Science and Engineering, Sichuan University, Chengdu 610065, China*
** Corresponding author: Han Liu (happylife@ucla.edu)*

S1. Mean ± Standard Deviation Plot of Atomic Displacement over Time

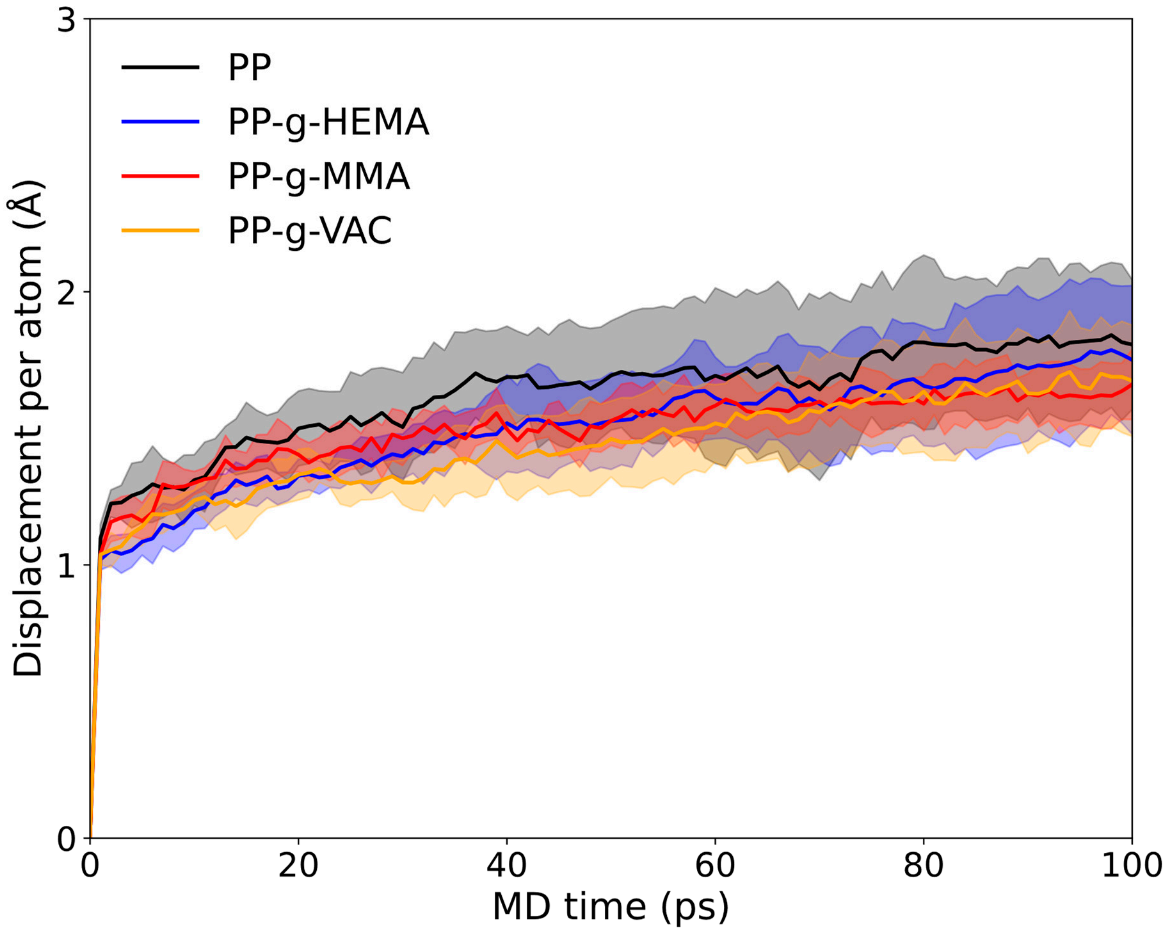

Figure S1: Mean ± standard deviation plot of atomic displacement over time, as a reference to the max–min boundary plot of Fig. 8B in the main text.
